# Psychological perspectives on robotic assisted pivotal response treatment in autism

**DOI:** 10.1038/s41598-025-27616-3

**Published:** 2025-12-15

**Authors:** Gema Benedicto-Rodríguez, Pedro Bellon-Berna, José Manuel Ferrández

**Affiliations:** 1 Department of Electronics, Computer Technology and Projects, Polytechnic Universidad Politécnica de Cartagena, Cartagena, Spain; 2 ECTLab, European University of Technology, Cartagena, Spain; 3 Institute of Bioengineering, University of Miguel Hernández de Elche, Elche, Spain; 4 Universidad Politécnica de Cartagena, Cartagena, Spain

**Keywords:** Neuroscience, Psychology, Psychology

## Abstract

The impact of Pivotal Response Treatment (PRT) assisted by the social robot Pepper on the emotional regulation and treatment adherence of children with autism spectrum disorder (ASD) and their caregivers is explored. Given the integration of technology and psychology, our study provides empirical evidence on the role of social robots not only as therapeutic tools but also as facilitators of family engagement and personalized ASD interventions. Our findings highlight significant improvements in children’s emotional responses with the use of a social robot and offer new insights into the variability of caregiver adherence.

## Introduction

ASD is a neurodevelopmental condition rooted in neurobiology^1^, characterized by challenges in social cognition and communication, restricted interests, and repetitive behaviors^2^. Symptoms of ASD, such as social withdrawal, limited social initiative, hypoactivity, and a lack of emotional modulation, typically emerge within the first year of life^3^ according to the diagnostic criteria outlined in the *Diagnostic and Statistical Manual of Mental Disorders*,* Fifth Edition* (DSM-5)^4^, that categorizes ASD into three levels of severity based on the degree of support required^5^. Understanding the roots of ASD is complex, involving genetic and environmental factors affecting brain development. Despite extensive research, its exact causes remain elusive, highlighting the need for improved diagnostic methods and deeper understanding of ASD’s mechanisms.

Providing appropriate intervention approaches for young children with ASD is crucial for achieving optimal developmental outcomes^6^. A wide range of therapies has been implemented to improve communication, reduce sensory issues, and enhance learning in children with ASD. Among the most widely used are behavioral interventions based on the principles of Applied Behavior Analysis (ABA), including structured methods such as Discrete Trial Training and more naturalistic approaches like PRT^6^.

ABA is a well-established and extensively researched approach that uses learning principles to teach socially significant behaviors and reduce problematic ones. It targets domains such as attention, imitation, cooperation, communication, and language^7^. However, traditional ABA programs often involve intensive schedules—ranging from 20 to 40 h per week—which may be emotionally demanding for both children and families^8,9^. Critiques have also pointed to issues such as rigidity in program design^10^, challenges in skill generalization^11^ and reduced intrinsic motivation^12^.

PRT, while grounded in the principles of ABA, differs significantly in its focus and implementation. Rather than concentrating on isolated behaviors, PRT targets “pivotal” areas of development—such as motivation, responsivity to multiple cues, self-initiation, and self-management—believed to produce widespread improvements across multiple domains^13^. By using the child’s natural environment and interests as the foundation for learning, PRT fosters engagement and facilitates the generalization of skills to real-world contexts^14^. It also reduces stress and increases enjoyment for both children and caregivers when compared to more rigid ABA models^15,16^.

Family involvement is a core component of PRT, empowering parents to implement strategies across daily routines and thereby improving consistency and outcomes. Nonetheless, this involvement also requires time and dedication, which may pose practical challenges for families^17^. The convergence of home-based strategies and larger interventions shows the possibility of applying psychology and incorporating technological tools.

In line with this interdisciplinary perspective, a growing trend has emerged towards the combination of diverse disciplines, such as psychology and technology, from which various technological devices emerge^18^. In search of effective components of interventions for children with ASD, the use of technology such as robots has received increasing interest over the last decade^19,20^. Recent comprehensive analyses confirm that robots are particularly effective in teaching social skills in ASD populations through structured, repetitive, and highly engaging interactions^21^.

In contrast to other technologies, robots provide embodied multimodal features (speech combined with gestures and other movements) that are important when training social communicative behavior. Robots are appealing to many children with ASD and may contribute to a more positive effect^22^ and may be useful in showing higher behavioral predictability through simplified social uses and more repetition^23^.

One such technological tool is Pepper, a humanoid social robot designed to interact dynamically with humans. Equipped with facial and emotional recognition capabilities, Pepper communicates through conversation, gestures, and its touchscreen interface. Though its functionality is relatively basic, Pepper has found applications in corporate and educational settings. Importantly, it can be programmed using *Choregraphe* software to align with PRT methodology, making it a promising candidate for psychoeducational interventions aimed at children with ASD^24^.

The characteristics of Pepper directly support the objectives of this study. Its ability to recognize emotions and provide consistent, multimodal interactions aligns with the goal of evaluating the emotional impact of robot-assisted interventions on children with ASD. Additionally, its programmability for PRT allows for structured and personalized therapeutic sessions, facilitating the assessment of adherence to treatment and the involvement of primary caregivers.

## OBJECTIVES

Building on the growing integration of technology in various sectors, including the field of psychoeducational interventions, this study aims to explore the potential benefits of incorporating social robots in the context of children with ASD. With the aim of improving the design of interventions to make them more effective and personalized, information is provided on the effect of the implementation of a social robot, called Pepper, in psychoeducational interventions on the emotions of children with ASD and the improvement in the characteristics associated with ASD.

The secondary objectives are listed next:

1. To examine the variability in the emotions of children with ASD before and after the intervention sessions in the robot and instructor group.
2. To assess the perceptions of the primary caregivers about the emotions of the children in the robot and instructor group.
3. To study the children’s adherence to treatment, in the group with a robot and with an instructor.
4. To analyse the adherence maintained by the main caregivers, analysing the strategies learned and applied in the family environment, in the group with robots and with the instructor.
5. To investigate the effect of the children’s demographic characteristics (age, IQ, sex, severity of ASD symptoms and psychiatric comorbidities on all of the above-mentioned aspects.
6. To encourage the active participation of the main caregivers through session rating questionnaires, gathering feedback on their experience in robot and instructor groups.
7. To study the score obtained from the Social Communication Questionnaire (SCQ) to observe the evolution of the presence of the traits associated with ASD, in the group with robot, with instructor and control.
8. To examine the commitment/involvement of the main caregivers to the therapeutic intervention through each of the previous points, in the group with robot, with instructor and control.

As the control group only completed the SCQ questionnaire and did not attend the intervention sessions, some secondary objectives are limited to robot and instructor groups, while SCQ-related objectives include all three groups.

## METHODOLOGY

The study included participants diagnosed with ASD, according to the child’s adaptation to human-robot interaction in the initial first contact, prior to therapy. All the participants were divided into 3 groups. The assignment of the different participants to each group was made according to several criteria, such as a balanced number of participants in each group (n) and diversity of demographic characteristics and severity of ASD symptoms (see Table 1), while also taking into account each individual’s preferences as much as possible. The final groups were:

**Table 1:** Individual characteristics of the two groups of participants: PRT + Robot, and PRT + Instructor.

Groups	Children	Age	Sex	TIQ	Severity of ASD symptoms	Psychiatric comorbidity
PRT + ROBOT	Child 1	6	M			
	Child 2	7	M	91	1	Language delay, paroxysmal non-epiletic episode, toilet incontinence and maintenance insomnia
	Child 3	10	M	81	2	Oppositional defiant disorder, Asperger’s syndrome, ADHD
	Child 4	10	M		1	
	Child 5	11	M	139	1	
	Child 6	11	M			
	Child 7	11	M		1	Attention deficit disorder, conciliation insomnia
	Child 8	11	M	80	1	Accompanying language impairment and learning difficulties
	Child 9	12	M	57	2	Delayed language and inattention. Intellectual deficit and accompanying language impairment
PRT + INSTRUCTOR	Child 10	5	M	92	2	Language impairment
	Child 11	6	M	119	1	Language impairment
	Child 12	7	M		2	Accompanying intellectual deficit and accompanying language impairment
	Child 13	7	M	84	1	Delayed maturation
	Child 14	8	M	110	1	
	Child 15	8	F			
	Child 16	8	M	81	2	Conduct disorder
	Child 17	9	M	75	2	ADHD Sleep disorder

- Group 1: Robot-assisted PRT + Instructor (*n* = 9). The participants in this group received PRT performed by a robot.
- Group 2: PRT with instructor only (*n* = 8). This group received PRT in a traditional way.
- Group 3: Control group (*n* = 8). The participants in this group did not receive any specific PRT-related items during the study. It was included for comparative comparisons and to evaluate the effects of the interventions in the other two groups.

The control group only answered the SCQ questionnaire, as they did not participate in the intervention sessions. For this reason, their results are only shown in the figures corresponding to SCQ.

### Eligibility criteria

The final requirements to participate in the study: to have an up-to-date clinical diagnosis of ASD based on the criteria established by the DSM-V and supported by psychological tools such as the Autism Diagnostic Observation Scale (ADOS-2) or Autism Diagnostic Interview-Revised (ADI-R), among others. The age of the participants must be between 5 and 12 years of age, along with the use of at least one word accompanied by gestures.

To check the effectiveness of the robot and the treatment, it is necessary not to have previously received PRT and in the case of taking medication, the instructor must be informed. As long as participants meet all the requirements established so far, an intelligence quotient (IQ) of 70 or higher will not be a restriction.

### Intervention protocol

The robot and instructor groups consisted of 12 sessions of 20 min for each group, with 2 sessions per week.

To obtain an adequate number of participants and ensure the diversity and representativeness of the sample, which is crucial for the validity of our research results, the sample of participants was obtained through direct contact with schools and autism associations in the city of Elche, by the researcher of the study. Among the entities that collaborated were AITEAL, Mentes Divergentes, Soles sin Voz and Colegio Jorge Guillén.

Sample size was determined by the availability of participants who met the criteria for inclusion in local autism associations. Although this limits the generalizability of the results, it provides valuable information about the applicability of the intervention in a real, community context. The small sample size is due to several factors such as the personalized nature of interventions for children with ASD, the specific characteristics of the individuals involved, and the need for carefully managed, human-directed research. In addition, it is important to note that the study involves minors who meet specific criteria, and the number of participants with ASD depended on the geographic area in which the study was conducted.

Subsequently, these centers are informed about the requirements that must be met to participate in the intervention program, and the parents are given an informative letter with consent to authorize the child’s participation in the study. The program is developed by a certified PRT researcher and was approved by the Research Ethics Committee of the San Juan de Alicante University Hospital, with code 22/033 and was carried out in strict accordance with relevant guidelines and regulations. All the procedures used complied with the established ethical and scientific regulations, guaranteeing respect for the principles of integrity and well-being of the participants.

After the first contact with the robot took place and the participant was classified into a certain group of the study, a training talk on PRT was held for parents where they were provided with information on the steps to follow at home, ending with the delivery of a questionnaire to understand the PRT method.

Days before the start of therapy, participants must use the Even Better virtual platform, where, through simple games, basic emotions and how to identify them are explained. In this way, when starting the sessions, the child will recognize the characters of said platform and can choose how they feel before and after each session, represented in an *Emodiana*.

### PRT implementation

PRT implementation strategies are key to increasing its effectiveness. These include first securing the child’s attention, providing clear and appropriate opportunities, choosing activities that motivate them, rotating new tasks with skills already mastered to gain confidence, and immediate and natural reinforcement of the activity and even when they have tried with effort^14,25^.

To implement these strategies, the robot follows the following steps: (1) the robot moves in such a way that the child receives an incorrect signal when deviating from the target behavior, (2) the robot asks open-ended questions, and (3) creates an opportunity for the child to respond, and (4) the robot acts accordingly by providing reinforcement.

### Assessment tools

**Initial PRT Questionnaire and Tips (Initial)**.

Particularly those with ASD, present a unique challenge due to the limited availability of validated self-report tools. Traditional instruments such as the Self-Assessment Manikin (SAM) and Premo demand significant cognitive effort, making them less suitable for children with language or emotion recognition difficulties^26^.

**Fig. 1:**
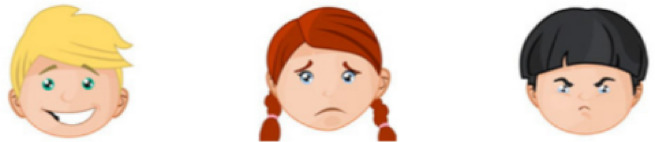
Core emotions: happiness, anger and sadness represented in the adapted Emodiana.

For this reason, we used the *Emodiana*, a scale based on 10 emotions with optimized graphic design and an emotional target that measures intensity through colors^27^. Although the original *Emodiana* scale comprises ten emotions^27,28^, in this study we used an adapted version specifically designed for young children with ASD. The adapted scale focused on three core emotions (happiness, anger, and sadness) (Fig. 1) represented by facial icons from the Even Better digital platform, which children were already familiar with.

At the beginning and end of each session, children were asked to select the face that best matched how they felt at that moment.

**Fig. 2:**
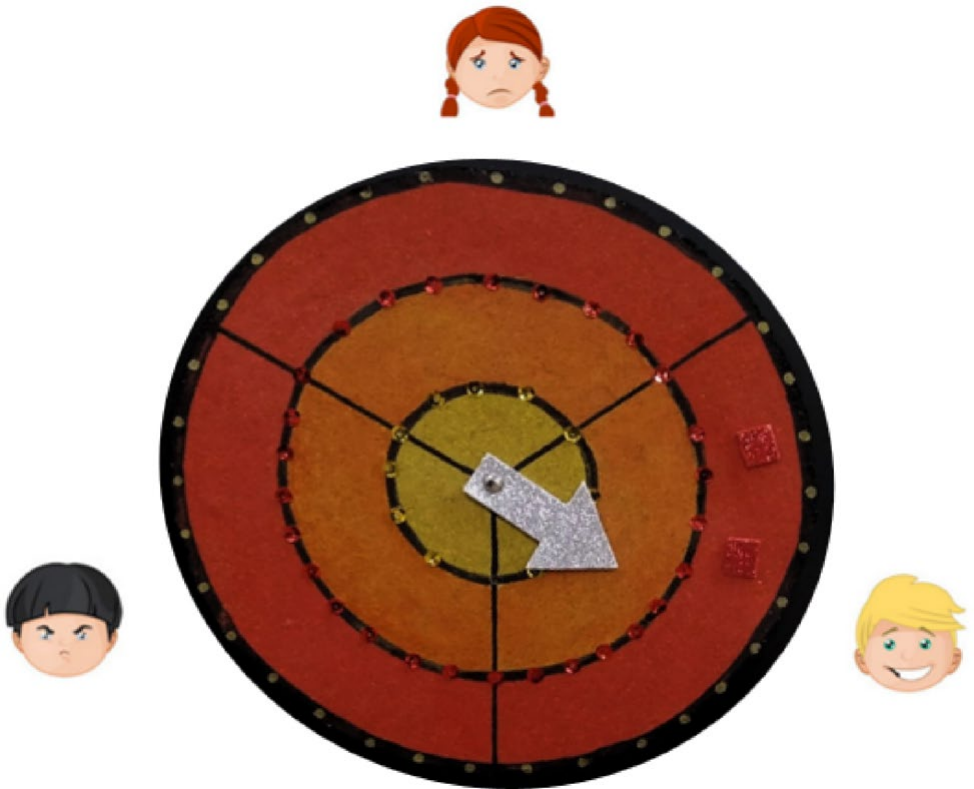
Adapted Emodiana scale used in this study (happiness, anger, sadness), with the 3-point color-coded intensity system (yellow = low, orange = medium, red = high).

Each selected emotion was scored using a color-coded intensity scale (1 = yellow, 2 = orange, 3 = red) (Fig. 2). Higher scores reflected stronger affective intensity, typically associated with happiness. This adaptation ensured reliability, reduced cognitive load, and improved ecological validity for participants as young as 4–5 years old.

**Children and Parents Emotions Questionnaires (Start and End of Each Session)**.

At the start of each Session both the children and their parents must do a questionnaire. The parents fill out a short questionnaire with two sections: first, they select from a range of emotional expressions to show how they think their children feel before and after the session; second, they specify the reason behind the chosen emotion.

**Session Qualification (During Each Session)**.

Originally, this was a 10-item measure using a Likert-type scale; however, it was modified into a four-item version using a Visual Analogue Scale (VAS) to enhance internal consistency and address validity concerns^29^. This adjustment has been supported by numerous studies that underscore the advantages of VAS in various contexts^30,31^.

**Adherence Questionnaire (End of Each Session)**.

This evaluation aims to determine whether parents are adhering to the instructions of PRT as part of the study. The key questions guiding the assessment include whether all PRT treatment guidelines have been followed since the last visit, whether at least three treatment strategies have been implemented, and whether the guidelines have been applied consistently throughout the day or only during specific moments. Additionally, the evaluation seeks to identify any challenges parents have faced in following the guidelines and whether they would recommend the treatment to others.

Likewise, the *Emodiana*, Session Rating and Adherence questionnaires were selected for their applicability in contexts of emotional and psychotherapeutic intervention. Although these tools have not been specifically validated for their exclusive use in children with ASD or in robot-assisted interventions, their use has been previously documented in clinical and educational contexts with the child and adolescent population, showing acceptable psychometric properties. *Emodiana* has been used as an emotional self-assessment tool adapted for children and adolescents in school and clinical contexts^27^. The Session Rating Questionnaire has proven to be useful for collecting participants’ impressions of the therapeutic experience^32^, while the Adherence Questionnaire is based on scales already used in therapies aimed at children and young people^22^. The choice of these questionnaires responds, therefore, to their accessibility, suitability to the profile of the participants and history of use in related research.

**Social Communication Questionnaire (End of Each Session)**.

It was applied to observe the evolution of the presence of the traits associated with ASD, in the group with the robot, with instructor and control.

Caregivers completed the Social Communication Questionnaire (SCQ), a validated screening instrument specifically designed to identify the core symptoms of Autism Spectrum Disorder through of 40 dichotomous (Yes/No) items derived from the ADI-R, and evaluates three core domains associated with ASD: (1) social interaction difficulties, (2) communication impairments, and (3) restricted, repetitive, and stereotyped patterns of behaviour. The current study employed the Current Form (Form B), which assesses the child’s behaviors over the last three months, making it appropriate for detecting short-term improvements associated with robot-assisted and human-led interventions^33^.

Both parents were invited to complete the SCQ independently each week. In most cases only one parent responded, but in some cases, both did, and in those instances both responses were retained in the analysis to capture inter-informant variability.

**Fig. 3:**
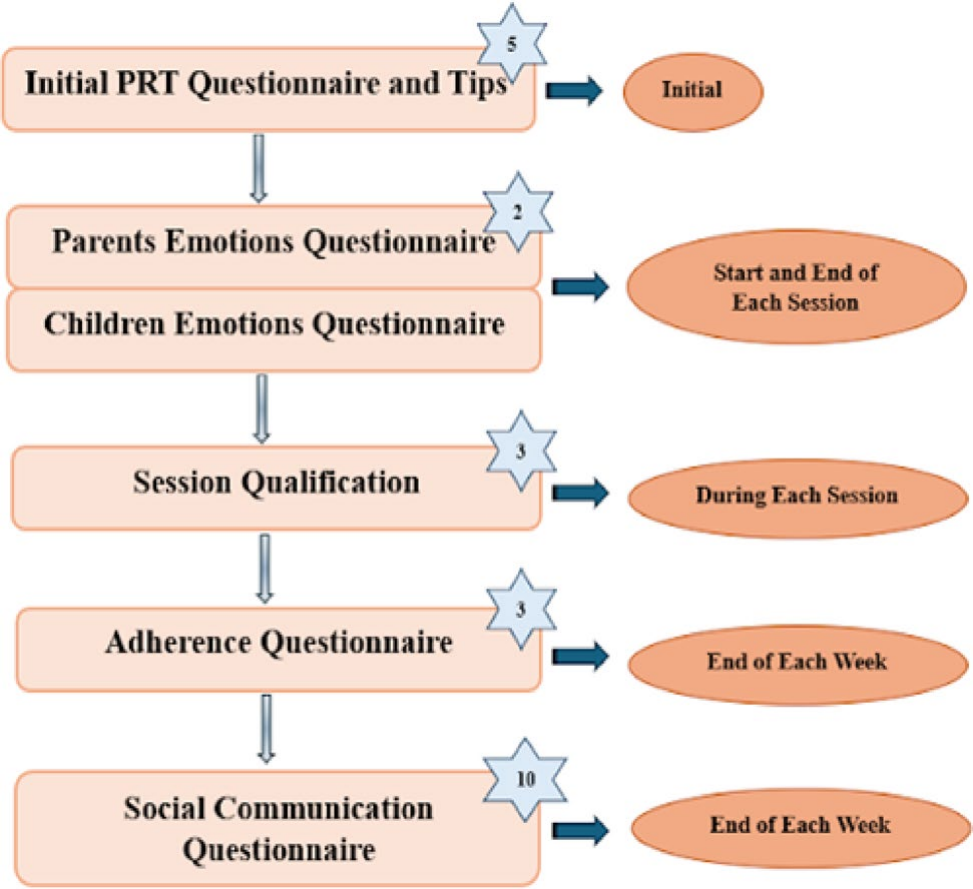
Flowchart illustrates the process of implementing various psychological tests throughout the study and the duration in minutes of each test (stars).

The order in which the different psychological tests were implemented can be seen in Fig. 3.

## RESULTS

The participants can be sorted according to different aspects in Table 1 [Insert Table 1 here]:

- **Age** There are two groups: the first group of 6–10 and the second group of 11–12 years. Both groups male gender.

- **Intelligence Quotient (IQ)** There are three groups: the first group below average: <90, the second group on average: 90–110, and the third group above average: >110.

- **Severity of ASD symptoms** There are two groups according to the severity of features in grade 1 vs. grade 2.

- **Psychiatric Comorbidity** There are four groups: group 1 (language delay, accompanying language impairment, inattention, and intellectual deficit), group 2 (maintenance insomnia and conciliation), group 3 (Oppositional Defiant Disorder and Attention Deficit Hyperactivity Disorder (ADHD), and group 4 (other disorders).
  The control group is not included in Table 1 because these participants did not take part in the intervention sessions and were not analyzed in terms of age, IQ, ASD severity, or comorbidities. Their contribution was limited to completing the SCQ questionnaire on a weekly basis, and their data are therefore only presented in the figures and analyses related to the SCQ.

**Emotions Questionnaire**.

**Fig. 4:**
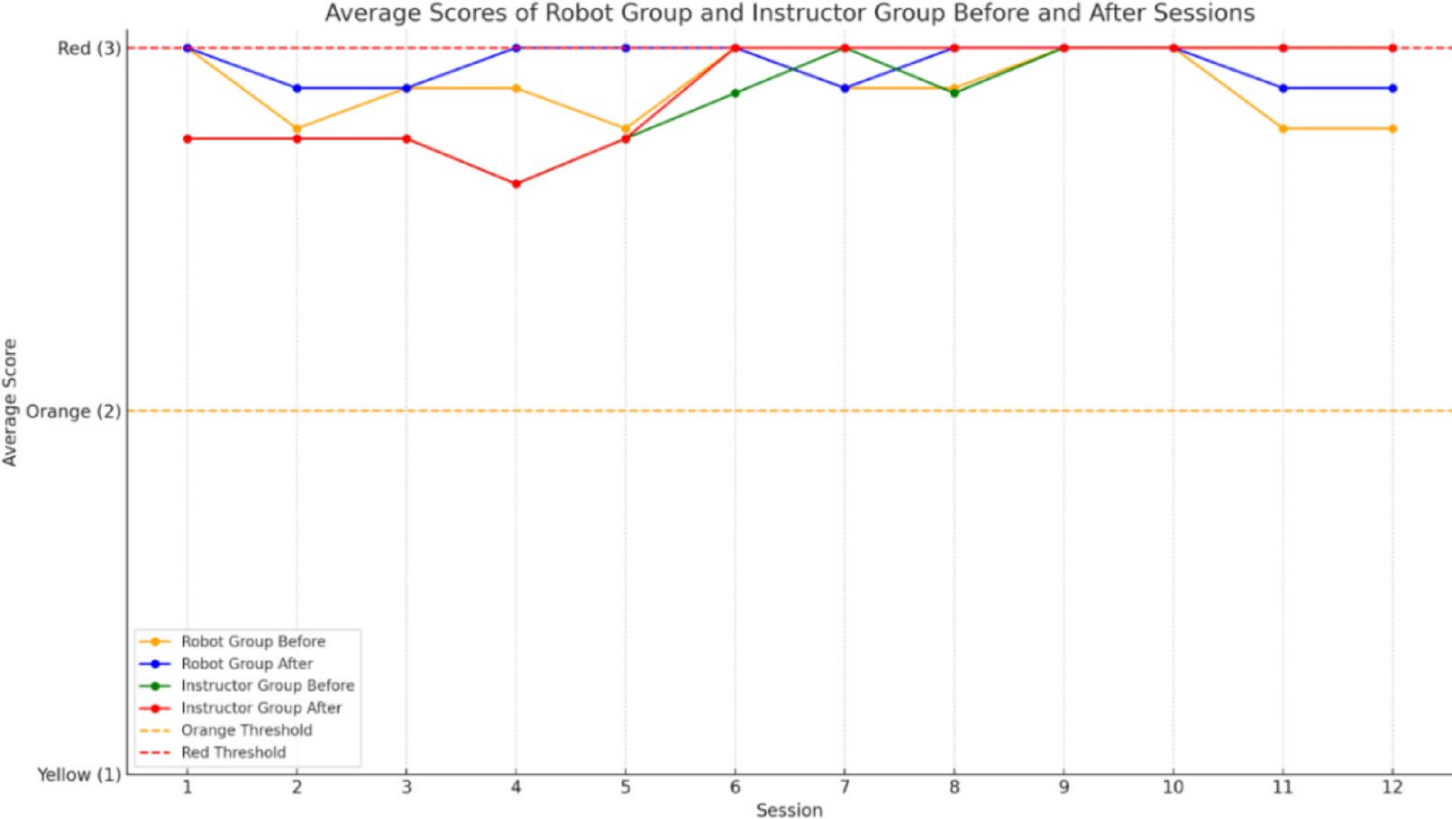
Average scores of children on the Emotions Questionnaire before and after sessions in robot and instructor group, categorized by emotional intensity (Red = 3, Orange = 2, Yellow = 1).

In the *Emodiana* tool that has been created in this study, the scores depend on the colors chosen by the participants, from yellow representing 1 point to red representing 3 points, i.e. the maximum score. Figure 4 shows the average emotion scores of the children who participated in groups 1 and 2. The highest scores throughout the 12 sessions indicate more positive emotions and those that are lower, more negative emotions, represented by colored lines: orange (before) and blue (after) group 1, green (before) and red (after) group 2.

With respect to group 1, the scores are mostly 3.00, both before and after the sessions, as is the case in group 2. For group 1 there is a drop in scores in session 7 but throughout the sessions the scores are similar. For group 2 there is a drop in the first 5 sessions both before and after the sessions, although from session 6, in both cases they increase until they reach the maximum score that remains stable.

In the first sessions, as indicated by the score threshold, the scores in both groups are below the threshold, that is, not so positive emotions, although as the sessions go by, the scores in both groups increase.

To determine if there were significant differences in both group 1 (with a robot) and group 2 (with an instructor), the Student’s T-test was performed. For group 1 the results show a t-statistic of −2.72 and a p-value of 0.0076, and for group 2 the results show a t-statistic of −1.0 and a p-value of 0.3506. As for the first group, the null hypothesis is rejected, i.e., it indicates a significant difference between the values before and after robotic therapy. Compared to the second group, there are no significant differences between the scores before and after the sessions.

For group 2 (with instructor) it cannot be separated by age, because all participants belong to the same group. When calculating the averages of the responses to the emotion target of children in IC therapy, the Kruskal-Wallis statistic is 14.94 and a p-value of 0.00057 before the sessions, which indicates a significant difference, as well as after the sessions in which the statistic is 13.07 and the p-value 0.00145. These significant Kruskal–Wallis results in Group 2 correspond to the comparison between IQ subgroups (< 90, 90–110, > 110), as defined in Table 1.

Regarding the degree of involvement before the sessions, the Kruskal-Wallis statistic is 0.093 and a p-value of 0.7609, and after the sessions, the Kruskal-Wallis statistic is 0.394 and a p-value of 0.5300. In both cases, there are no significant differences.

Finally, with respect to the comorbidities present, valid comparisons between the groups cannot be made due to the presence of a single participant in some of the categories.

**Fig. 5:**
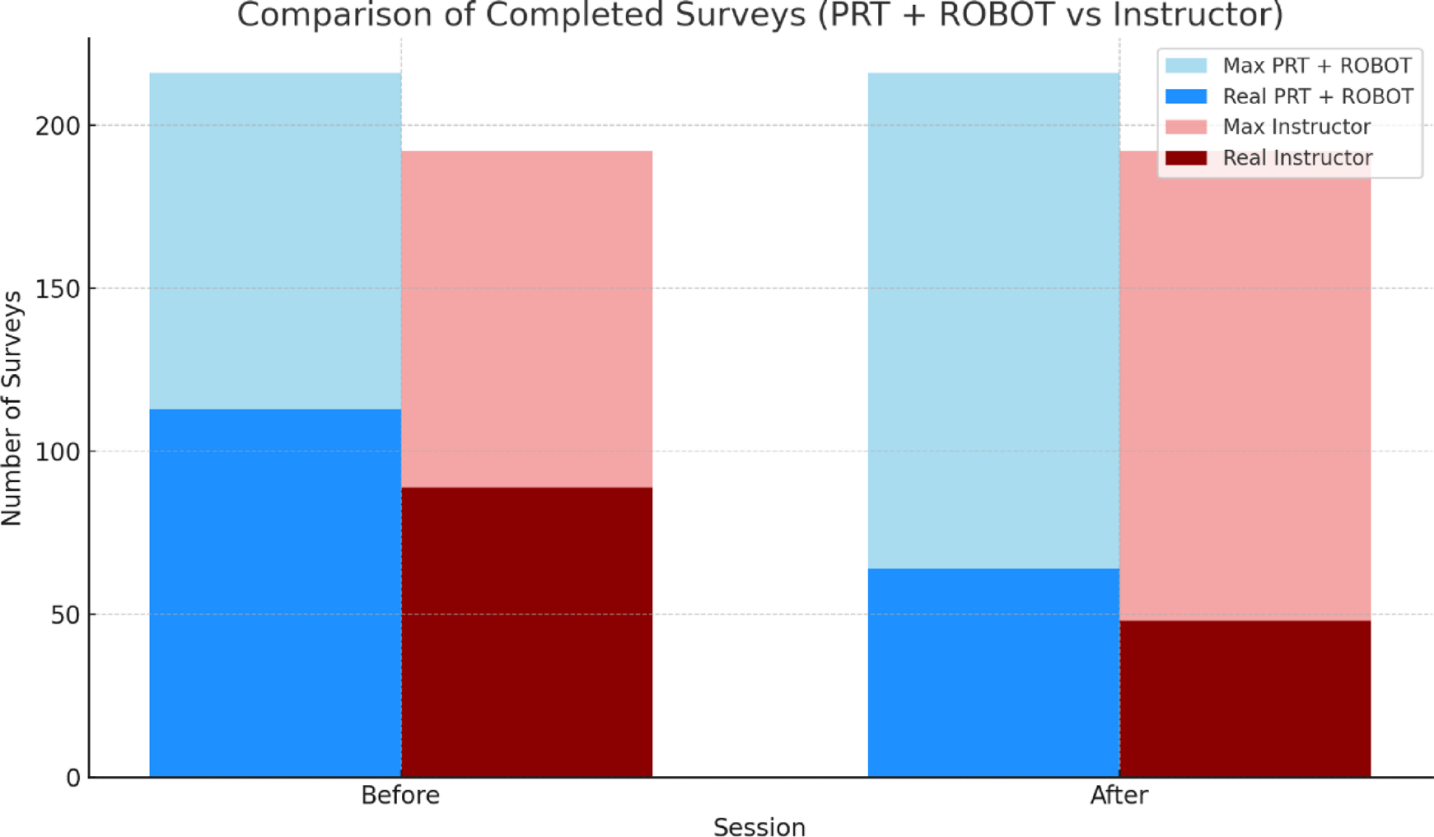
Comparison of the maximum number of possible surveys completed by parents on the Emotions Questionnaire in the robot and instructor groups.

Regarding the participation of parents or primary caregivers in the Emotions Questionnaire, Fig. 5 shows the significant difference between the number of surveys completed by group 1 (before session 113 and after session 64) and group 2 (before session 89 and after session 48) with respect to the number of surveys that should have been completed throughout the entire treatment period (216 before and after the session).

**Fig. 6:**
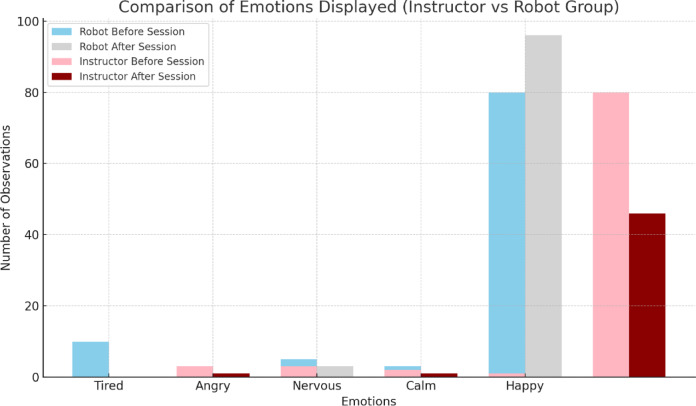
Comparison of emotions displayed by parents before and after session in robot and instructor groups.

With respect to the emotions of the participants in the robot group perceived by their parents before entering the session and at the end of it, a sample of “happy” responses is collected throughout the therapeutic process in its entirety, compared to the other emotions, as is the case with the group with an instructor (Fig. 6). The results are presented separately, as the response categories are specific to each type of intervention.

**Fig. 7:**
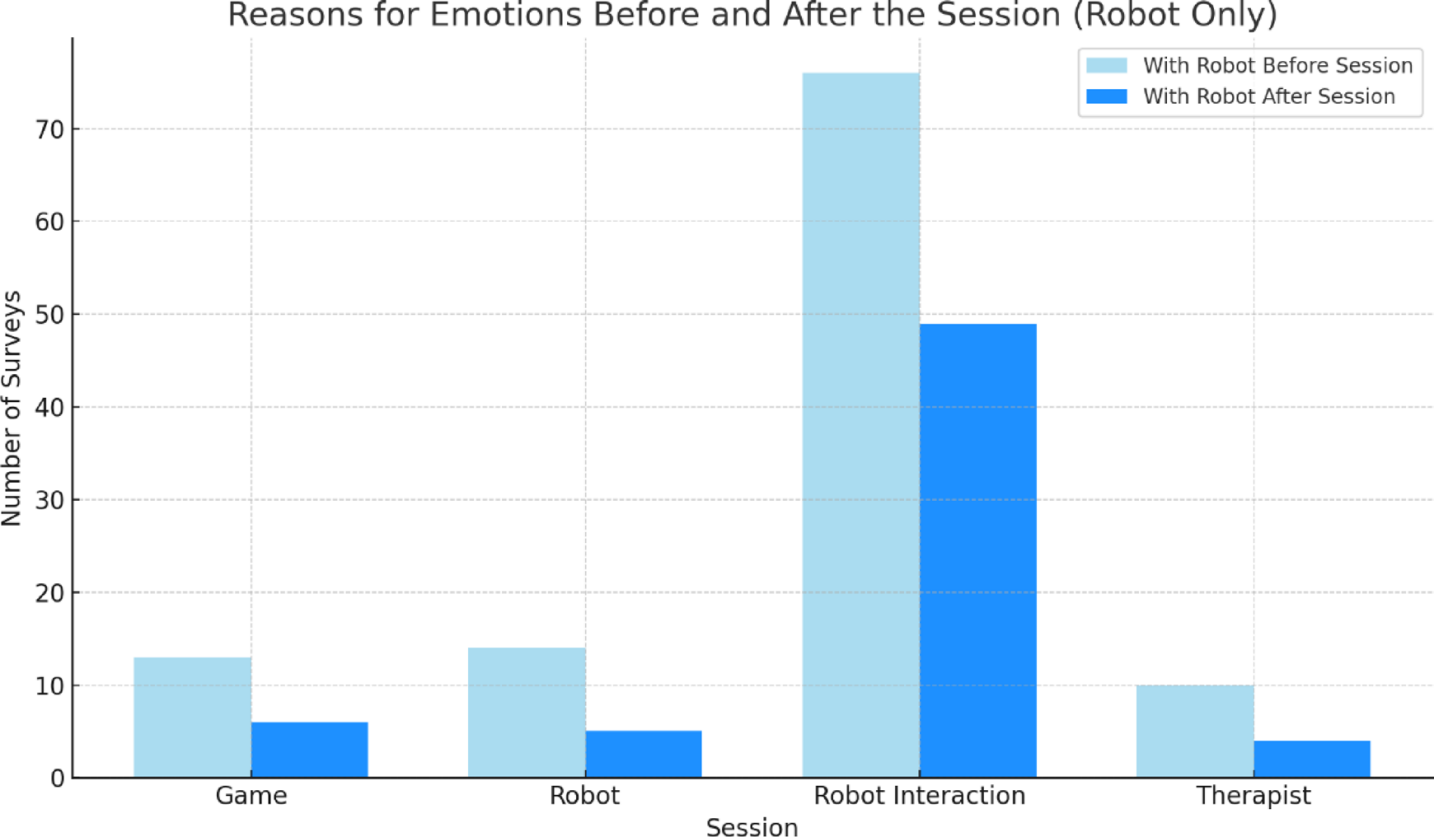
Reasons for emotions of the children before and after session in the PRT + ROBOT.

**Fig. 8:**
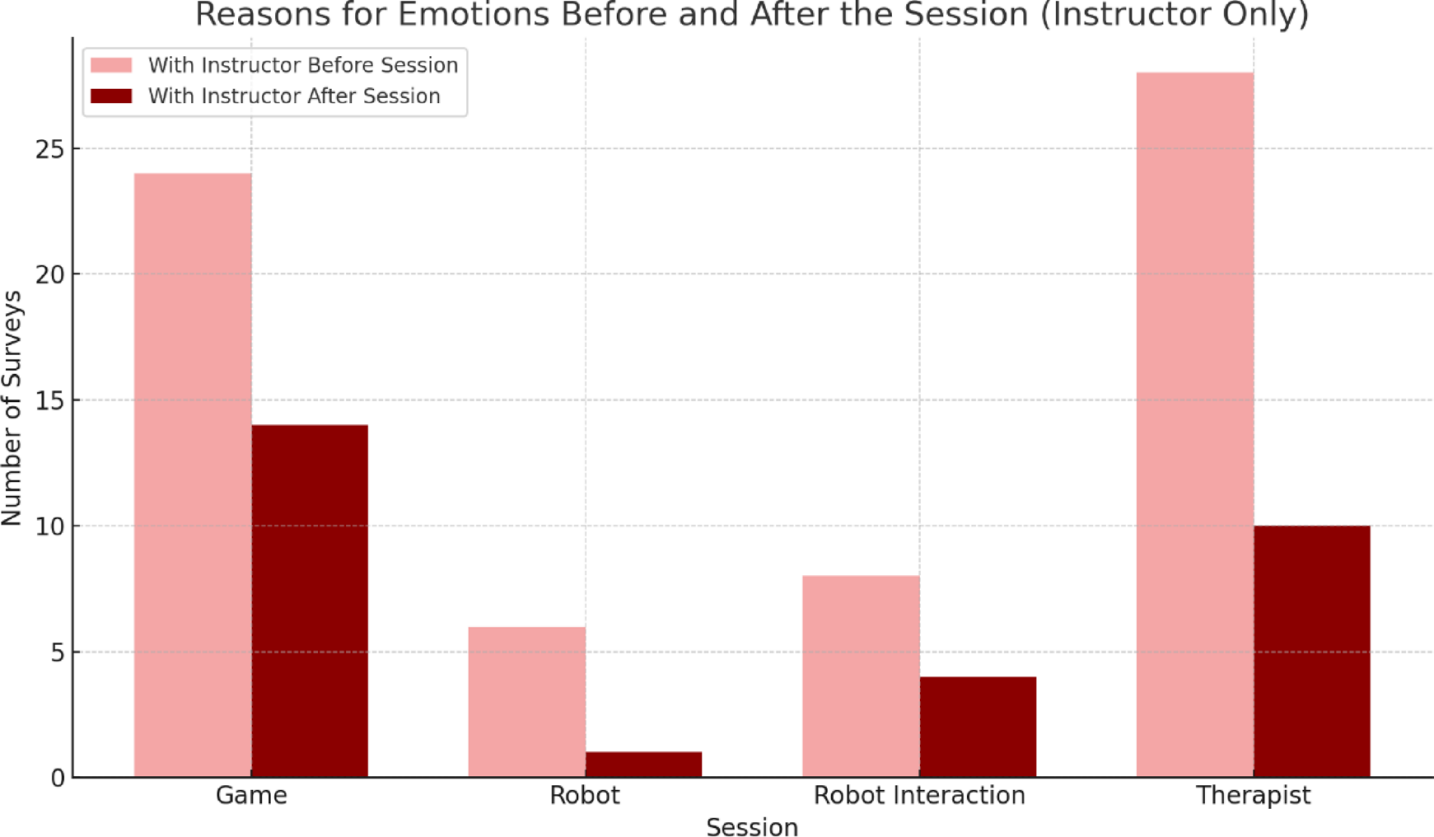
Reasons for emotions of the children before and after session in the PRT + INSTRUCTOR.

Figure 7 shows the reason why parents consider these emotions to be due, and that is the interaction of the robot during the different games in the group of sessions with the robot, which is maintained after the sessions. With the instructor (Fig. 8), emotions are more balanced, although with a greater number of surveys related to the presence of the therapist and the game. It is understandable that the interactions with the robot have less weight, since in the sessions it was only about me and should have a score of 0. After the sessions, the emotions related to the game remain high. In both conditions, most caregivers perceived their children as ‘happy’ both before and after the sessions, reflecting a ceiling effect on this measure. In the group with an instructor, some answers were heterogeneous and even the robot was mentioned as the cause of the emotions, despite the fact that it was not present, which shows some confusion in the completion of the questionnaires.

**Session Qualification (During Each Session)**.

**Fig. 9:**
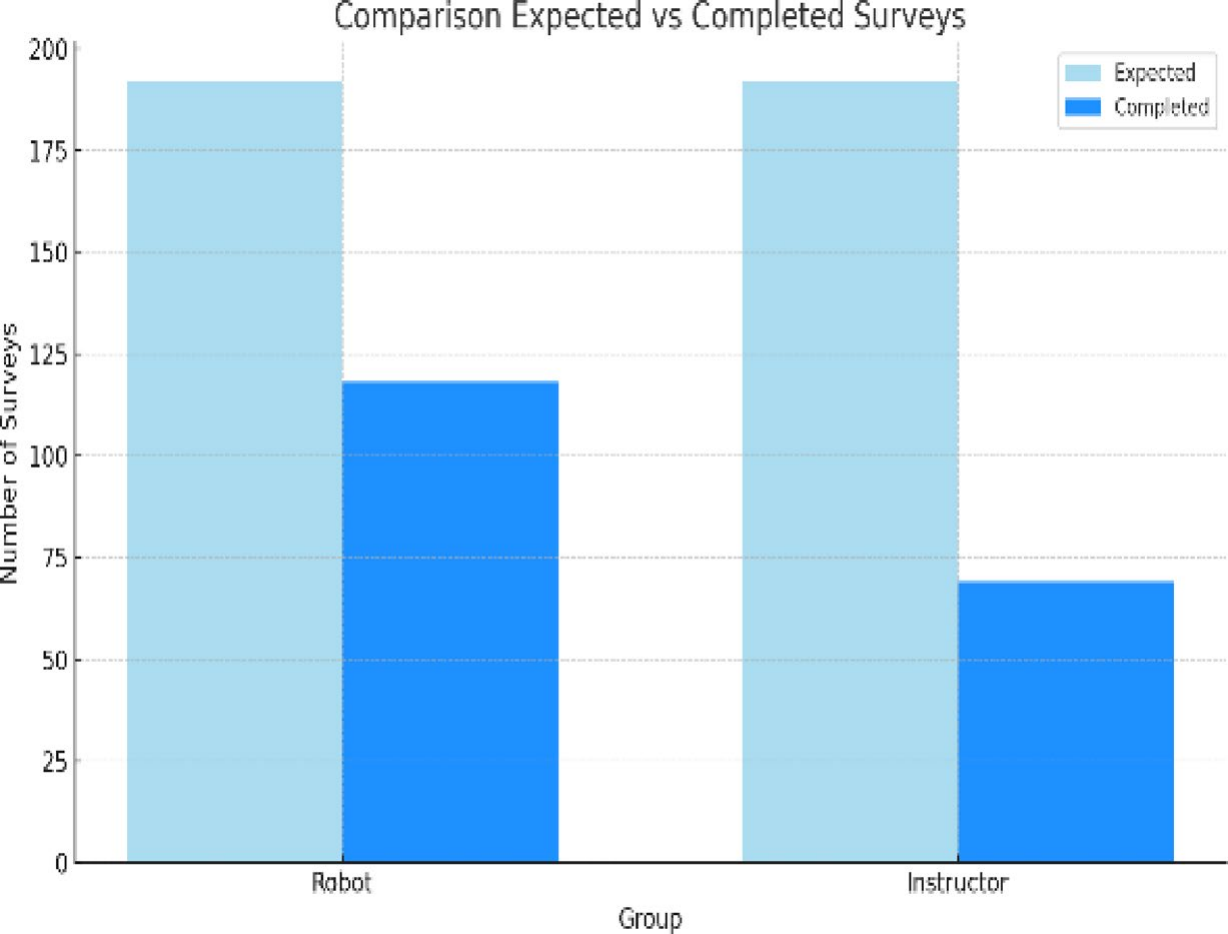
Comparison of the maximum number of possible surveys with the total number of surveys completed by parents in the session qualification questionnaire.

To assess the degree of satisfaction with the therapeutic process, both in the group with robot and with the instructor, the session rating questionnaire was applied, from which only 118 responses were obtained out of the 192 expected (including father, mother or main caregiver) for the group with robot, and 69 answers out of the 192 expected for the group with instructor. as shown in Fig. 9.

**Fig. 10:**
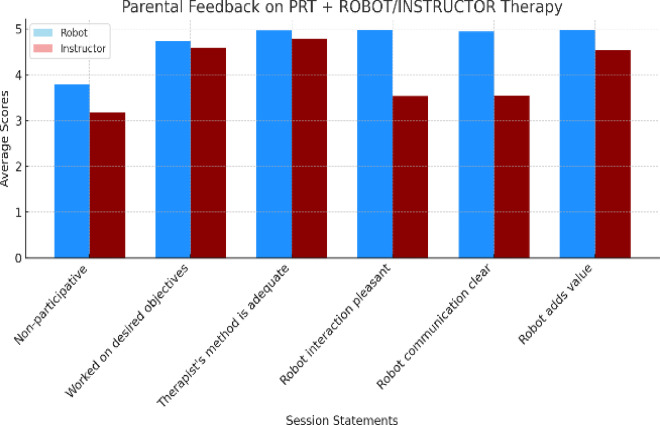
Average rating to parental adherence to robotic therapy in the session qualification questionnaire measured on a scale of 1 to 5.

As indicated in Fig. 10, in the answers obtained to each of the six questions, robot group shows higher scores, especially for statements like “Worked on desired objectives” and “Robot adds value,” indicating positive feedback. On the contrary, instructor group shows slightly lower scores in comparison, particularly in statements about participation and aspects related to the robot.

**Adherence Questionnaire (End of Each Session)**.

The mean adherence of the group to the robotic-assisted PRT protocol was 91.7%, although when classified by categories, three participants showed complete adherence (33.3% over 100%) to the study, while five participants showed incomplete adherence: three participants in one session (33.3% over 91.7%) and three participants in two sessions (33.3% over 83.3%). While, in the group with an instructor, out of 8 participants, 6 attended all the sessions, obtaining 100% adherence to the study, except for two participants who represented 91.67% attendance and 83.33% attendance. In the latter case, he attended the sessions but did not want to carry out the activities or collaborate, having to suspend the sessions. The mean adherence of the group with the instructor was 96.88%.

The causes of non-compliance in the sessions, both for the robot and instructor group, are the interruption of the use of the robot due to technical problems with the robot’s software and with other devices such as the E4 *Empatica* bracelet or webcam, loss of interest in the game scenarios, exhaustion and the effects of a change in medical treatment.

After calculating the standard deviation of adherence in the robot group, both individually and as a group, the threshold of outliers was determined, with a lower limit of 79.2%. With these data, none of the participants had a percentage of adherence lower than 79.2% (the lowest percentage being 83.3%), so it is considered that there are no outliers, that is, that the adherence to treatment of any participant is below the standard deviation of the mean. For the group with an instructor, the individual standard deviation of the attendances is 5.80% and for the group 6.20%. Having a lower threshold of 87.57%, no outliers are found.

With respect to the robot group, the results in Table 1 suggest that age and degree of ASD influence adherence to sessions, while IQ and psychiatric comorbidities do not show a significant effect. As for the group with an instructor, the results suggest that there are no statistically significant differences between the groups according to IQ, the degree of ASD and possible comorbidities, that is, these aspects do not influence adherence to therapy.

Similar to the adherence of the group of children with robots, the parents showed an adherence classified by the participants as: child 1 (father 8.33% and mother 91.67%), child 2 (father 16.67% and mother 83.33%), child 3 (father 16.67%, mother 58.33% and sister 25%), child 4 (father 16.67% and mother 66.67%), child 5 (father 100% and mother 100%), child 6 (father 41.67% and mother 41.67%), child 7 (father 16.67%, mother 75% and grandfather 8.33%), child 8 (mother 83.33% and grandmother 16.67%) and child 9 (mother 75% and stepfather 25%).

The adherence of the parents or main caregivers of the group with instructor is: child 1 (father 33.33%, older sister 100% and mother 66.67%), child 2 (father 66.67% and mother 33.33%), child 3 (father 33.33% and mother 66.67%), child 4 (father 16.67% and mother 83.33%), child 5 (older brother 25%, father 16.67% and mother 83.33%), child 6 (mother 100%), child 7 (66.67% and grandmother 33.33%) and child 8 (father 25%, little sister 25% and mother 100%).

In the group of participants with robots, the standard deviation of 28.41% shows a high variability in the attendance of parents and caregivers, while, in the group with an instructor, the standard deviation of 4.23 indicates a moderate variability, reflecting an imbalance in the participation of the family environment.

**Fig. 11:**
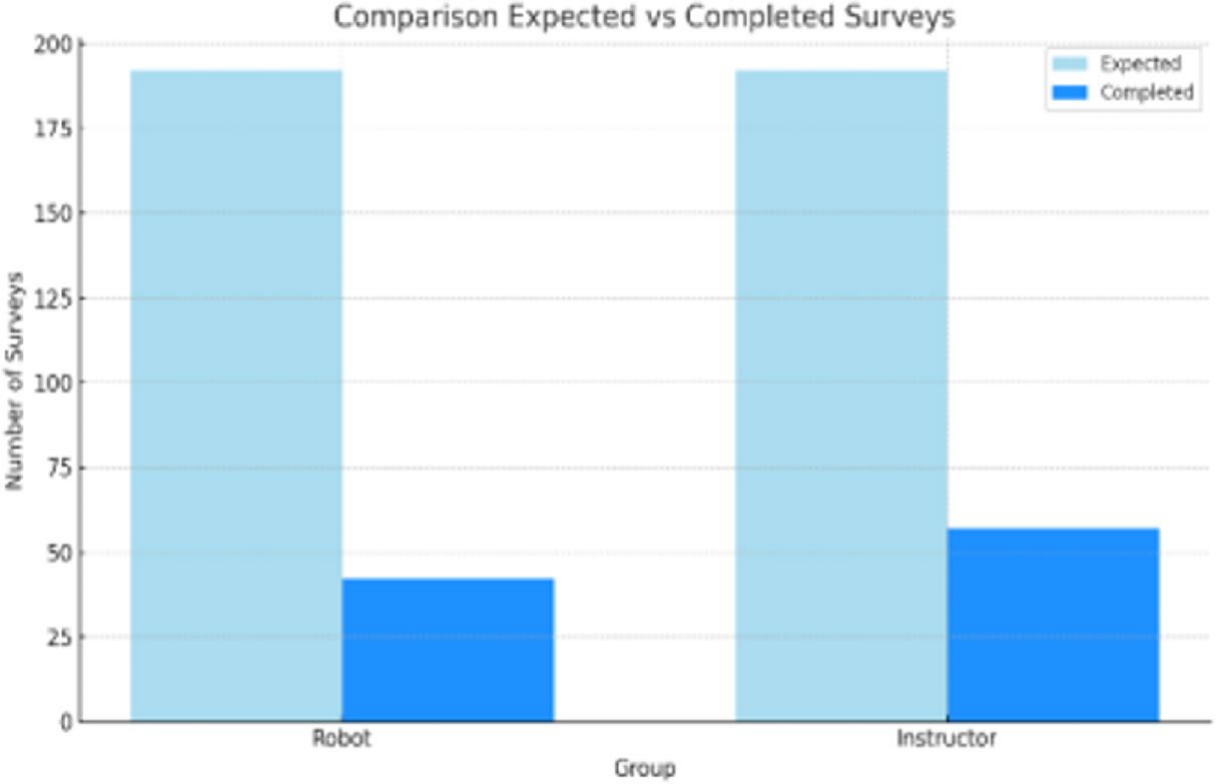
Comparison of the maximum number of possible surveys with the total number of surveys completed by parents in the adherence questionnaire.

To determine whether adherence or participation in the study was maintained in the family, an adherence questionnaire was administered, from which 42 responses were obtained out of the 108 expected (including father, mother or main caregiver), which demonstrates low adherence to treatment (38.89%), as shown in Fig. 11, as well as 57 responses out of the 192 expected in the group with instructor (29.69%).

**Fig. 12:**
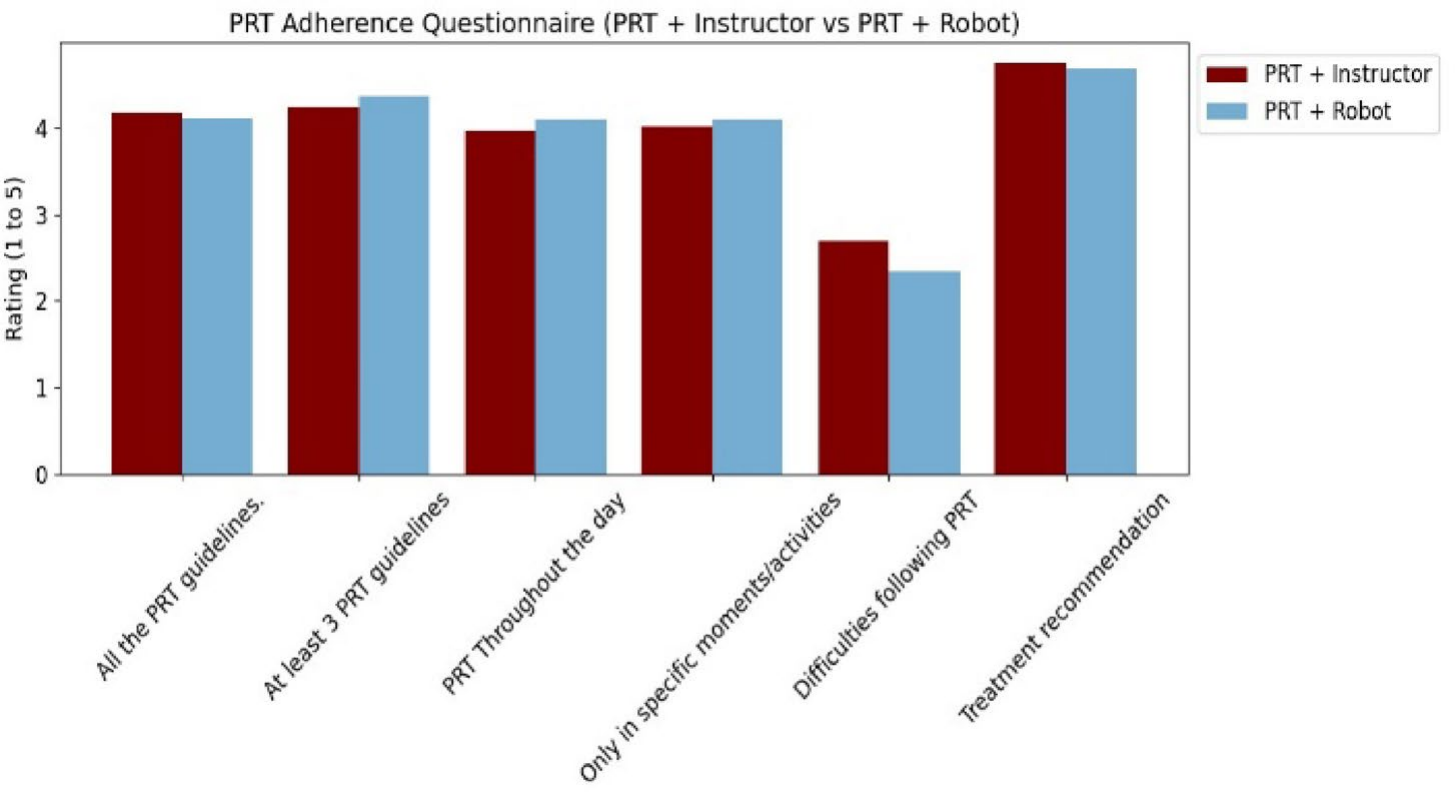
Average rating to parental adherence to robotic therapy in the adherence questionnaire measured on a scale of 1 to 5.

As indicated in Fig. 12, the answers obtained from the six questions that make up the questionnaire of the robot group show similar results in most categories, although with some areas that are rated higher, such as difficulties in following PRTs and specific activities. In the group with an instructor, they tend to show slightly higher scores, especially with regard to adherence to the guidelines and the recommendation of therapy.

**Social Communication Questionnaire (End of Each Session)**.

**Fig. 13:**
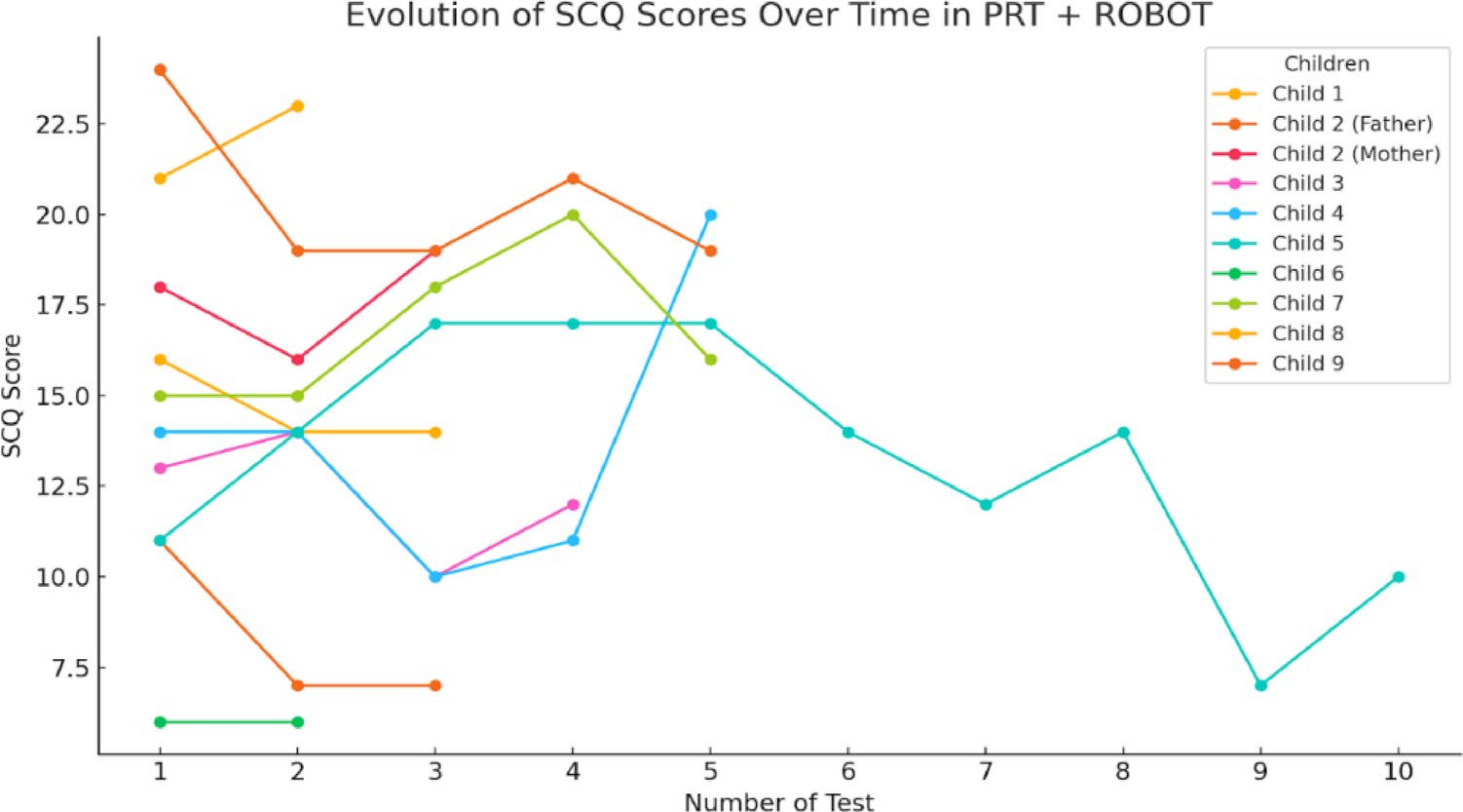
Evolution of the SCQ scores obtained during 12 sessions in the PRT + ROBOT group

When analyzing the general patterns of the different groups of participants, it can be seen in Fig. 13, with the PRT + ROBOT group, that most of the children show fluctuations at the beginning, although later they tend to stabilize. The increase is gradual, they improved as the sessions progressed. However, there are exceptions such as child 1 who shows a sharp increase and decrease, while child 9 has a more stable increase in scores towards the last sessions. Child 4 has a drop in scores, especially towards later sessions, which could indicate a decline.

As shown in Fig. 13, for one child both mother and father provided independent responses. Both series are presented in the figure and included in the analyses, since they reflect different perspectives on the child’s functioning.

**Fig. 14:**
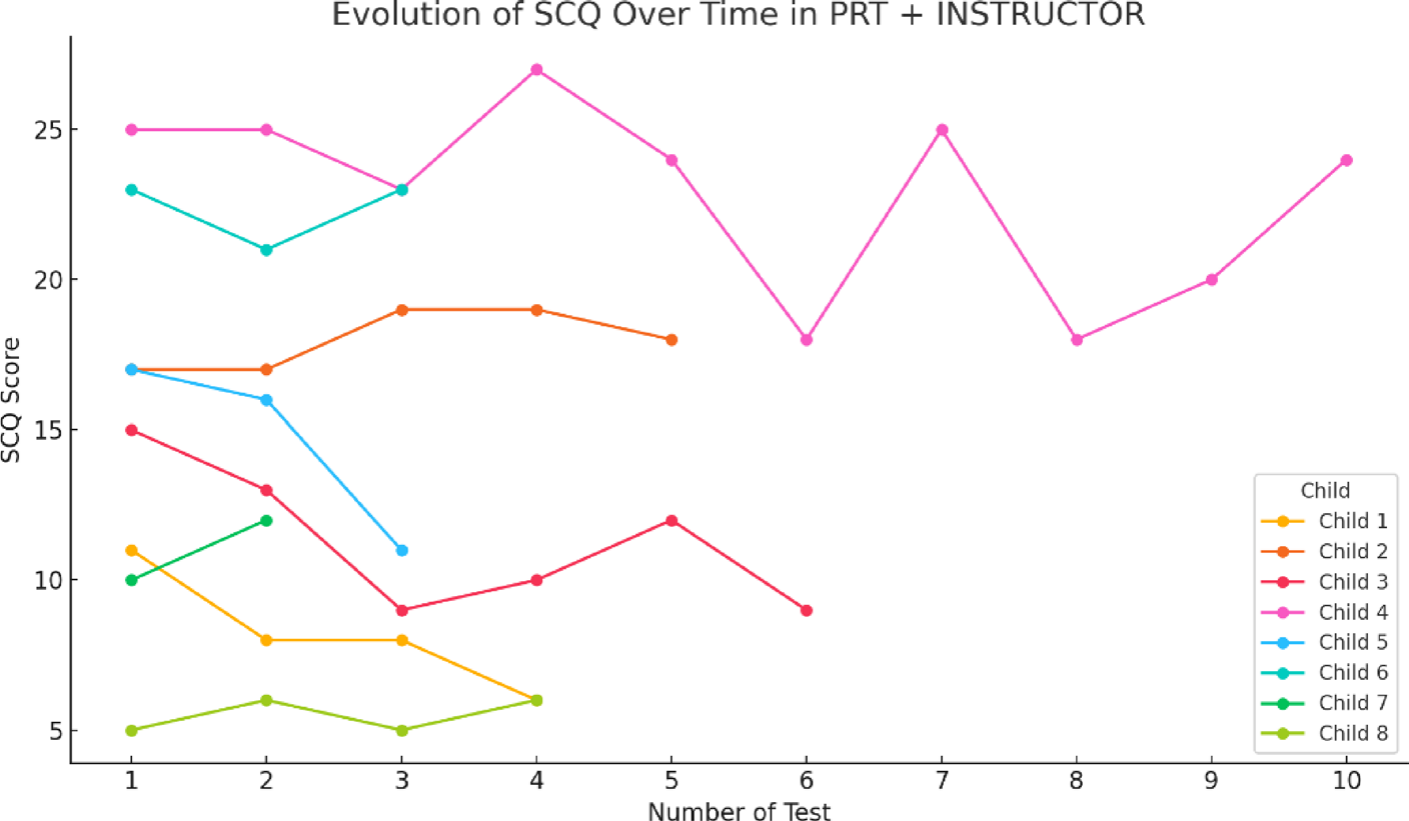
Evolution of the SCQ scores obtained during 12 sessions in the PRT + INSTRUCTOR group.

As for the PRT + INSTRUCTOR group, the results are similar to the first group, i.e., there are fluctuations, but they show a gradual increase. However, other children have more drastic changes, suggesting varied experience and less persistent progress (Fig. 14).

**Fig. 15:**
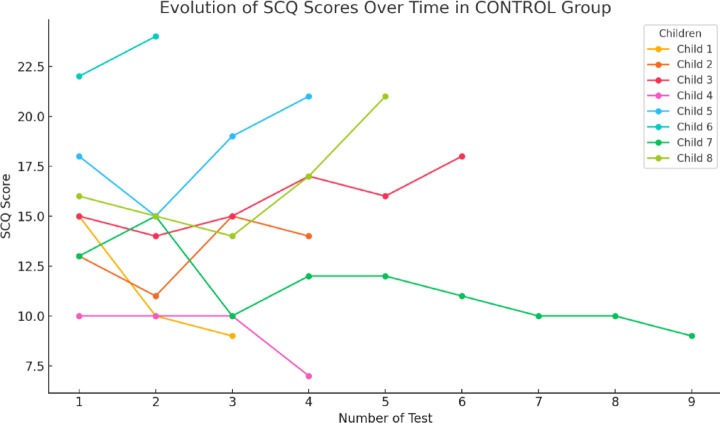
Evolution of the SCQ scores obtained during 12 sessions in the PRT + CONTROL group.

Finally, in Fig. 15 the results of the CONTROL group show increases, many changes and instability or downward trends. This indicates that they did not experience as much progress as the rest of the groups.

## DISCUSSION

The analysis of the results obtained in this study reveals significant differences in children’s emotions depending on whether therapy sessions were conducted with a robot (group 1) or a human instructor (group 2). The children’s emotions showed a positive trend as the sessions progressed, particularly in the group interacting with the robot. This phenomenon could be related to increased familiarity and acceptance of the robot over time, as noted in previous research. For instance^34^, concluded that repeated interactions with social robots could improve children’s mental well-being, while^35^ attributed such improvements to the phenomenon of familiarization.

Robotic- and human-instructor-assisted teaching in participants with autism exhibits similarities in effectiveness when structured protocols are applied, making it easier for both approaches to improve social and communication skills. However, robots offer a more predictable and less emotionally overwhelming environment, which reduces anxiety and allows greater engagement in some children. In addition, they are usually more effective in specific tasks, such as joint attention and the personalization of activities. On the other hand, human trainers can instantly adapt strategies, address complex social nuances, and maintain long-term interests, especially if flexibility is required^36^.

Statistical analysis revealed significant emotional differences between the groups, with a more pronounced impact in the robot group (*p* = 0.0076) compared to the instructor group (*p* = 0.3506). This finding supports the hypothesis that interaction with robots has a differential effect on children’s emotions^37^. highlighted that the Pepper robot can act as a communicative facilitator, promoting autonomy and emotional development in children with ASD.

Another noteworthy aspect was the higher caregiver participation in the robot group, driven by the implementation of the Session Rating Questionnaire at the end of each session. This instrument provided valuable feedback on the therapeutic experience and helped identify areas for improvement, strengthening collaboration between caregivers and the therapeutic team. However, it was observed that some caregivers in the instructor group answered questions related to the robot’s characteristics or actions, which did not align with the conditions set for that group. This could reflect a lack of attention to or understanding of the activities conducted during the sessions, emphasizing the need for clearer protocols to ensure comprehension of each intervention’s context. Additionally, this result might stem from the initial curiosity often sparked by innovative technologies, as suggested by^38^. Conversely^39^, noted that robotic technologies promote greater engagement by offering interactive and dynamic experiences, which, in turn, enhance caregivers’ involvement in the therapeutic process.

Caregivers’ adherence to strategies learned during therapy, reinforced through feedback collected in the Session Rating Questionnaire, is crucial to maximizing the treatment’s benefits. This approach reinforces skills practiced in sessions, identifies barriers within the family environment, and facilitates the generalization of skills to everyday contexts, as highlighted by studies like^40^. However, the emotional burden experienced by caregivers can compromise their ability to implement these strategies consistently. This phenomenon suggests that the stress associated with caring for children with ASD may reduce adherence to recommended practices, limiting the intervention’s potential benefits. Therefore, it is essential to consider caregivers’ emotional state and external demands when designing and evaluating therapeutic interventions, as their effective participation is a key factor in the emotional and behavioral progress of children.

Regarding specific emotions, children in the robot group showed a more pronounced perception of positive emotions such as “happy.” This effect may be attributed to the robot’s ability to capture and sustain attention through interactive games, a finding consistent with the work of^34^, who found that social robots are effective in assessing and improving children’s mental well-being. Other studies, such as the 2024 research from UPCT, highlight the role of the Pepper robot in reducing tension and promoting empathy in children with ASD^41^.

However, this study also had limitations. Factors such as comorbidities were not directly evaluated, which reduced the heterogeneity of the data but allowed for a clearer focus on the primary diagnosis, as suggested by^42^. Additionally, the lower number of surveys completed after the sessions might be related to caregiver fatigue or limited availability, a phenomenon already identified by^43^ in the context of prolonged therapies. Including both parents’ responses when available may have introduced additional heterogeneity, but we considered it important to retain all informants in order to reflect the ecological validity of caregiver perspectives. Future studies could analyze parent reports separately to explore inter-informant differences more systematically.

These results should be interpreted with caution, given that the caregivers’ perception had a generalized ceiling effect and, in the case of the group with an instructor, included less consistent responses (for example, attributing the emotion to the robot in sessions where it did not participate). This may limit the reliability of the measurement and reinforces the need for more objective complementary assessments (e.g., trained external observers or physiological records) to be used in future studies.

The emotional patterns indicate that children in the robot group associated their positive emotions directly with the interaction with the robot, while in the instructor group, emotions were more evenly distributed between interaction with the therapist and play. This finding suggests that robots may play a specific role in emotional dynamics, whereas human instructors offer a more balanced and personalized experience, aligning with the observations of^19^. These results underscore the importance of considering the particularities of each approach when designing therapeutic interventions for children.

In evaluating the level of satisfaction with the therapeutic process in the robot and instructor groups, as measured by the Session Rating Questionnaire administered at the end of each session, significant differences were observed in participants’ perceptions. In the robot group, a higher number of responses (118) were recorded compared to the instructor group (69), out of a total of (192) expected responses per group. This higher level of participation in the robot group could be attributed to the interest, novelty, or curiosity generated by technology, as social robots often capture greater attention and foster more active engagement in therapeutic contexts^44^. support this hypothesis, highlighting how interactive AI-based agents used in neurorehabilitation training enhance user engagement through novel and customizable interactions.

Furthermore, higher scores in the robot group on aspects such as goal achievement and the added value of technology indicate a positive perception of its effectiveness and relevance. It should be noted that aspects such as curiosity, motivation, novelty effect, goal achievement, or added value of technology were not directly measured in this study. They are discussed here as interpretative insights, in line with previous literature on technology-mediated interventions. This could be due to the perceived impartiality and consistency associated with robots, which fosters greater trust in the therapeutic process. According to^45^, robots can provide predictable and judgment-free interactions, features that facilitate engagement in therapeutic settings. These terms should therefore be understood as theoretical interpretations intended to contextualize our findings, rather than as empirical outcomes of the assessment tools used.

On the other hand, the instructor group showed slightly lower scores, which could be influenced by participants’ prior expectations. Human interaction often depends on subjective factors such as empathy or perceived affinity, which vary widely. Additionally, when directly comparing the two modalities, the instructor group may have been perceived as less dynamic or innovative. A recent study by^46^ suggests that empathy toward robots can influence the attribution of mental states to them, highlighting that human-robot interactions are perceived differently from human-instructor interactions, affecting the perception of empathy and affinity.

The positive evaluation of robots may also be linked to their association with innovation and modernity^47^. emphasize that the perception of robots as advanced tools capable of enhancing therapeutic processes generates a favorable predisposition, even before direct interaction occurs. This reinforces the idea that technological perception plays an important role in the evaluation of the therapeutic process, contributing to greater satisfaction and engagement in the robot group.

The analysis of results from the Social Communication Questionnaire identifies significant patterns in the progress of the evaluated groups. In the case of the PRT and robot group, a gradual increase in scores was observed, though initial fluctuations were present. This behavior aligns with studies supporting the effectiveness of Assistive Technology combined with PRT model^9,10^ known for its focus on key developmental areas such as communication and social skills. By utilizing natural reinforcements and sessions driven by the child’s interests, this approach enhances motivation and facilitates skill generalization to everyday contexts.

The implementation of key PRT strategies, such as capturing the child’s attention, interspersing new tasks with previously mastered skills, and using natural and contingent reinforcements, contributed to these advances. The robot’s ability to capture and maintain attention facilitated the application of these principles, ensuring effective learning opportunities while promoting motivation and persistence in tasks.

The initial fluctuations may be attributed to the novelty of the robot and the time required for children to adapt to this approach. Individual differences also play a crucial role, as evidenced by cases such as Child 1, who showed abrupt changes, and Child 9, whose progress was more stable. These variations reflect diverse responses to technological interventions, as discussed by^48^, who highlight that technology provides safe and predictable environments for training social skills, thereby enhancing PRT effectiveness. However, they emphasize that the success of these interventions depends on individual abilities and needs, reinforcing the importance of personalized strategies. Similarly^49^, note that initial interactions with robots are often inconsistent until a relationship of familiarity is established.

On the other hand, the PRT and instructor group also showed a gradual increase in scores, albeit with fluctuations and more drastic changes in some cases. This result aligns with studies emphasizing the fundamental role of human instructors in PRT-based interventions. Instructors have the ability to adapt their strategies in real-time, enabling more personalized progress but also introducing variability in responses, particularly due to differences in how children react to the instructor’s style.

The inconsistency observed in some cases could be related to interpersonal factors, such as the relationship between the instructor and the child, as well as the participant’s emotional state during the sessions^50^. emphasize that instructors’ ability to adjust strategies in real-time significantly influences children’s progress and the variability of their responses. Moreover^51^, underscore the importance of factors such as the instructor-child relationship and emotional state in the successful implementation of PRT.

In contrast, the control group showed a tendency toward instability and limited progress. This can be attributed to the absence of a structured intervention, such as the PRT model, which explains the lack of consistent improvements. The observed instability could be associated with the lack of consistent support, which is considered fundamental for fostering advances in social skills^40^. emphasize that children with ASD require specific, evidence-based interventions to improve their social communication. Similarly^52^, highlight that structured and specialized programs are more effective in improving skills and reducing symptoms, provided they are implemented appropriately.

These findings underscore the importance of designing personalized and structured interventions that account for both the individual characteristics of participants and the contextual factors influencing outcomes. They highlight the need for comprehensive and adaptive approaches in the development of social skills in children with ASD.

## CONCLUSIONS

The results of this study show that the intervention with a social robot (Pepper) and the intervention with a human instructor as a PRT model are effective in promoting positive emotions and progress in children with ASD, although the interventions are carried out through different mechanisms.

The robot intervention group shows a more stable and significant evolution in the emotions in the participants, probably due to the impact of the novelty, consistency and predictability of the robot. The intervention with an instructor, on the other hand, fostered a more adjusted and personalized experience, based on the therapeutic relationship and the ability to adapt at one time or another.

Participant adherence was high in both groups, although more variable in the robot intervention group. The participation of families, although based on the materials and resources used, was limited by factors such as fatigue or availability, which highlights the need to develop family support strategies.

The same study suggests that age and degree of ASD may influence children’s involvement, especially in technology-assisted interventions. The results suggest that the combination of technological tools and human accompaniment, as well as the adaptation of interventions to individual needs, can have very positive repercussions on the emotional, motivational and therapeutic dimension of children with ASD.

The present study has a number of limitations that should also be taken into account when interpreting the results. First, the small sample size, although within the usual range in similar ASD research. In turn, the symptomatological variability that characterizes ASD makes it difficult to generalize the findings to a wider population, and the personalization of the interventions performed makes this possibility of generalization even more difficult. Likewise, the engagement of participants and family members was affected, as the accuracy and reliability of their responses may be negatively influenced by possible fatigue or poor availability. The variability of the children’s responses and the limitations in the customization of the robots, since the programming of the robot was not adapted to the individual needs of each participant, but to the general protocol. may reduce its effectiveness. In addition, it should be considered in future studies that children with ASD have difficulties in expressing their emotions or thoughts, which implies that the evaluation of therapeutic progress depends on family members and can lead to a misrepresentation of the progress in the disorder.

Figure Captions.

## Data Availability

The datasets generated and/or analyzed during the current study are not publicly available due to ethical restrictions involving child participants, but are available from the corresponding author upon reasonable request.
